# Moderating Effects of Self-Esteem on the Relationship between Communication Anxiety and Academic Performance among Female Health College Students during the COVID-19 Pandemic

**DOI:** 10.3390/ijerph192113960

**Published:** 2022-10-27

**Authors:** Nisreen Al Awaji, Uzma Zaidi, Salwa S. Awad, Nouf Alroqaiba, Monira I. Aldhahi, Hadeel Alsaleh, Shahnaz Akil, Eman M. Mortada

**Affiliations:** 1 Department of Health Communication Sciences, College of Health and Rehabilitation Sciences, Princess Nourah Bint Abdulrahman University, Riyadh 11671, Saudi Arabia; nnalawaji@pnu.edu.sa (N.A.A.); hfalsaleh@pnu.edu.sa (H.A.); 2 Department of Health Sciences, College of Health and Rehabilitation Sciences, Princess Nourah Bint Abdulrahman University, Riyadh 11671, Saudi Arabia; uazaidi@pnu.edu.sa (U.Z.); emmortada@pnu.edu.sa (E.M.M.); 3 Department of Rehabilitation Sciences, College of Health and Rehabilitation Sciences, Princess Nourah Bint Abdulrahman University, Riyadh 11671, Saudi Arabia; sasahmed@pnu.edu.sa (S.S.A.); mialdhahi@pnu.edu.sa (M.I.A.); 4 Department of Laboratory Medicine, Clinical Physiology, Karolinska Institutet, SE-14186 Stockholm, Sweden; shahnaz_161@hotmail.com

**Keywords:** self-esteem, communication apprehension, academic achievement, COVID-19, female health sciences students

## Abstract

Unprecedented quarantine due to COVID-19 exposes individuals to withdraw from face-to-face interactions, which may influence communication and self-esteem (SE). Therefore, the overarching aims of this study are to examine the communication apprehension levels among female college students, and thus to investigate the moderating role of self-esteem on the relationship between communication apprehension and academic achievement. In this cross-sectional study, 287 female college students completed the survey, which was circulated through email. The survey included the following questionnaires: General Health Characteristics, Rosenberg Self-esteem Scale, and Personal Report of Communication Apprehension Scale. The results showed that 28.2% of participants were categorized as having a high level of communication apprehension, and only 9.8% had a low level of communication apprehension. The SE reported an overall score of 24.3 ± 2.14, indicating a high self-esteem level among students. The students’ grade point average (GPA) was positively correlated with SE. However, self-esteem as a moderator variable had no significant effect on the relationships between all predictors and GPA. The finding of the study highlights the need to implement different strategies to enhance students’ group discussions, meetings, and interpersonal communication to ensure the best learning outcomes. Future studies are required to investigate gender-based disparities in the relationship between communication apprehension and SE.

## 1. Introduction

The COVID-19 pandemic has affected individuals’ lives, influencing human beings in financial, physical, emotional, social, psychological, and moral ways. Social restrictions have caused communication and face-to-face interactions to be limited. During this time, many students experienced virtual education for the first time; consequently, the potential for communication issues and speech anxiety was raised.

Speech anxiety, also known as glossophobia, is the fear of public speaking. It may also be expressed as an individual’s cognitive anxiety when communicating with others [1]. In the literature, speech anxiety is also referred to as communication anxiety, communication apprehension (CA), and communication avoidance [2]. Studies have found that among university students, speech anxiety and various other psychological issues could be raised due to students’ limited engagement with their instructors and classmates through virtual classes during the pandemic [3,4]. Within this context, previous studies show an existing relationship between speech anxiety and student outcomes [5,6], as the former has been identified as a common pitfall to student success in learning [7,8]. 

In the literature, mixed results are available for undergraduate students’ academic achievement or performance during the COVID-19 pandemic. Some studies have reported enhanced self-efficacy, better research skills, and improved performance [9,10,11]. However, opposing studies exist. A survey of over 120 medical students from the Kirk Kerkorian School of Medicine in Las Vegas showed that, during the COVID-19 pandemic, first-semester students did not score above the national average as much as first-semester students before the pandemic [12]. Similarly, a study of over 200 students from Italian universities showed a negative impact of online learning on the concentration and learning abilities of students, two of the strongest predictors of poor academic performance [13]. Considering the above, it seems that student academic performance has been affected.

Aside from academic performance, many studies have assessed the psychological status of students. Here, as well, the results vary considerably, although most studies point toward increased anxiety and depression among students during the pandemic. The reasons behind the negative impact of online learning on psychological well-being are thought to come from lack of feedback, lack of opportunities for communication, cognitive load, technophobia, and reduction in enthusiasm [14]. According to a study conducted at different universities in Saudi Arabia, healthcare students had alarmingly high levels of depression [15], while students from Italy were also found to present depressive symptoms [13]. In a similar study conducted more recently, again in students from universities in Saudi Arabia, 75% of the students experienced some level of depression after being taught online for over a year [16]. 

Many factors can mediate or moderate the effects of negative consequences. Self-esteem is one of the psychological factors that plays a crucial contributing role in facing difficulties. It strengthens a person to use their abilities and mobilize personal resources while evaluating personal worth and value in a challenging situation [17,18]. In the literature, self-esteem has been studied in relation to students’ performance, interpersonal relationships, CA and lifestyle patterns [18,19]. The construct of self-esteem has been studied with various variables among health science students, including well-being and quality of life before the pandemic [20]. However, it will be interesting to investigate the moderating effects of self-esteem on CA and academic performance among health college students during the COVID-19 pandemic.

Only a handful of studies have assessed the self-esteem of students or their ability to communicate effectively with their peers or teachers. It is often neglected that teamwork, most frequently occurring in classroom sessions, boosts student engagement, improves communication skills, and enhances critical thinking abilities [21]. In support of this view, a recent study among dental students in the US reported that their academic performance may have decreased due to the lack of in-person group studying [22]. Similarly, according to the findings from a study from Kazakhstan, the students reported a negative impact on their communication and interpersonal relationships [23]. Another study related to the pandemic in the US posits that CA is elevated among students during online classes [6]. 

Aside from anxiety and academic performance, the impact of low self-esteem on academic performance has long been debated. Some of the evidence suggests that it may decrease one’s academic performance [24], although many argue that it may support academic performance [25]. A study conducted in Saudi Arabia showed that over 40% of university students experience low self-esteem [16]. Another study reported that online learning posed some behavioral challenges for them [11]. Importantly, it is well-established that self-esteem is negatively correlated with CA or apprehension [26]; that is, the cognitive anxiety an individual may experience when communicating with others [2]. Moreover, CA was found to be high among female college students as compared to male students [27,28]. It is crucial to assess the post-effects of pandemics on students’ communication, as prolonged isolation and living in continuous threat could negatively affect their performance [29]. 

If this is not addressed, it may lead to unresolved CA for many female students, which may eventually hurt their future success. There is still a dearth of studies assessing post-pandemic effects on communication patterns, self-esteem, and academic performance. Thus, the aim of the current study is to explore self-esteem, CA, and academic performance among undergraduate female students in health colleges during the COVID-19 pandemic and the possible correlation of CA with students’ learning outcomes. In the previous literature, CA was found to have a negative correlation with academic achievement [30]. Moreover, self-esteem was having a negative effect on CA [26]. Furthermore, low self-esteem causes a lack of interest in achieving high academic performance [31]. Therefore, it was hypothesized that “self-esteem will moderate the relationship between communication apprehension and academic achievement”. 

## 2. Materials and Methods

### 2.1. Study Design and Participants

A mixed-method design was used in which qualitative methods explained the quantitative method. The study was conducted during the COVID-19 pandemic between February and May 2022 at Princess Nourah University (PNU), KSA. A quantitative assessment of the students’ self-esteem and communication apprehension was carried out using a survey link with an invitation text, which was distributed through email and Twitter, to all female students enrolled in the 12 programs available at the College of Health and Rehabilitation Sciences (CHRS), PNU. The statistical package OpenEpi was used to calculate the sample size using an unbiased sample estimator approach [32,33]. A target sample size of at least 287 students was estimated based on a total number of 762 female students currently enrolled at CHRS, with a margin of error of 5%, a z score equaling 1.96, a confidence index level set to 95%, and a design effect of 3, given that non-probability sampling was applied. To ensure that a representative sample of students was collected from CHRS, the following equation was used to calculate the recommended number of students to be included in each of the 12 programs available at the college: (number of current students in program)/762 × 287. Thus, based on the calculation, the number of students to be recommended for inclusion in each program was recommended to be 42 for physical therapy (PT), 21 for occupational therapy (OT), 22 for speech and swallowing pathology (SLP), 22 for audiology (AUD), 32 for clinical psychology (CPY), 30 for epidemiology (EPI), 32 for clinical nutrition (CLN), 27 for health education (HE), 24 for diagnostic radiology, 8 for ultrasound imaging (US), 10 for radiotherapy (RT), and 17 for nuclear medicine technology (NMT).

### 2.2. Research Instrument

#### 2.2.1. Sociodemographic and General Health Characteristics with COVID-19-Related Information

The first part of the survey included 13 questions related to sociodemographic and general health characteristics, as well as COVID-19-related questions. For the sociodemographic characteristics, the participants were asked to report their age, specialty/program, level/semester that they had reached in the program they were enrolled in (Level 3 to 12), GPA in the last semester, marital status (single, married, other), and perceived economic status (low input, middle input, high input). 

For the general health characteristics, questions related to the participants’ general psychological health (absence or presence of psychological illness) and physical health status (absence or presence of health issues) were included. 

For COVID-19-related information, the participants reported their history of COVID-19 infection (absence or presence of history of COVID-19 infection), history of COVID-19 hospitalization for those who were infected (absence or presence of history of COVID-19 hospitalization), history of any immediate family members infected by COVID-19 (absence or presence of history of COVID-19 hospitalization), and, if applicable, relationship to infected family member.

#### 2.2.2. Rosenberg Self-Esteem Scale

The scale used to assess the students’ global self-esteem was the Rosenberg Self-Esteem Scale, which consists of 10 items that enable the assessment of positive and negative self-esteem [18]. The students’ level of agreement for each item was rated using a four-point Likert scale ranging from strongly disagree (1) to strongly agree (4). The translated Arabic version was used in the study to ensure a complete understanding of the items, and this version of the scale has reported high reliability (Cronbach’s alpha = 0.72) [20]. A reverse score was used for five of the items, and lower scores indicated lower self-esteem. A score < 15 was considered to indicate low self-esteem, as the average score was 15–25 points.

#### 2.2.3. Personal Report of Communication Apprehension (PRCA-24)

The scale used to assess the students’ concerns and feelings about communication with others encompasses four domains: group discussion, conversations in meetings, interpersonal discussions, and public speaking. This valid and reliable PRCA-24 was established and designed by McCroskey [34,35]. The translated Arabic version was used in the study to ensure complete understanding and has reported reliability (Cronbach’s alpha = 0.87) [36]. Each item’s level of agreement was rated using a five-point Likert-type format (1 = Strongly disagree and 5 = Strongly agree). The measure can range from a low score of 24 to a high score of 120, which indicates a higher level of apprehension. Additionally, the four domains (groups, meetings, interpersonal conversations, and public speaking) can range from a low score of 6 to a high score of 30. The cut-off points above 18 designate some degree of apprehension.

#### 2.2.4. Dual-Moderator Focus Group Process

In this study, one focus group was conducted and held in April 2022 at the College of Health and Rehabilitation Sciences in Riyadh. The purpose of this focus group was to obtain in-depth information on students’ perceptions of the impact of COVID-19 on communication patterns. Convenience sampling was used for the focus group. All the participants were from CHRS, representing four departments, and from the senior levels of bachelor’s programs. The focus group included six (6) participants. These participants were quantitative survey recipients too. Two trained moderators led the focus group. At the initiation of the session, the students provided written informed consent, and their rights were explained. Then, participant permission was granted, as the focus group was audiotaped and transcribed. Afterward, the students were informed about the purpose of conducting a focus group and had the ground rules explained to maintain confidentiality and smooth processing of the group discussion. Then, students were asked to complete a short questionnaire to record academic information (i.e., program, department, and level). These measures were conducted based on a focus group guide, and participants were encouraged to engage in a 90 min session.

#### 2.2.5. Focus Group Guide

The focus group aimed to describe the difficulties students face in communication after the COVID-19 epidemic. For this study, our questions focused on exploring student communication apprehension and operationalizing it so as relate to how much fear/anxiety a person feels in a range of contexts within an academic setting. The focus groups started broadly with questions about what contributed to students’ anxiety. Following this first set of questions, the moderator inquired about students’ anxiety when participating in class discussions, when conversing with a new acquaintance, and when giving a speech/presentation. Before ending the group, an exit question was asked, “Is there anything else you would like to say about post-pandemic CA?”

### 2.3. Statistical Analyses

The statistical analyses were performed using the IBM Statistical Package for Social Sciences (SPSS) for Windows, version 20 (SPSS Inc., Chicago, IL, USA). The internal consistency of the scales was calculated using Cronbach’s α coefficient. The normality of the data distribution was assessed using the Kolmogorov–Smirnoff test. Given that all data are not normally distributed, non-parametric tests such as Mann–Whitney and Kruskal–Wallis H tests were used, followed by post hoc testing with Dunn’s pairwise tests to investigate differences in the study variables (adjusted using the Bonferroni correction). Spearman’s correlation was used to examine the relationship between the study variables. 

Hierarchical moderated regression analyses were used, with GPA as the criterion variable and with the independent variables added to the model to determine the moderating effects of such independent variables by the change in the strength of the model. The moderator variable (RSE) was added to the second regression model. Then, the interaction term between self-esteem and PRCA was added to the third model. To avoid high multicollinearity with the interaction term, the variables were centered. Next, the interaction term between was added to the regression model. A ***p*** value ≤ 0.05 was considered statistically significant.

## 3. Results

Table 1 displays the sociodemographic characteristics of the respondents. Two hundred and eighty-seven students enrolled in the College of Health and Rehabilitation Sciences were included in the data analysis. The mean age of the participants was 20.81 ± 1.4 and their mean GPA was 4.48 ± 0.32. Respondents from the four departments were distributed as follows: 42.2% rehabilitation sciences, 27.2% health sciences, 16.4% health communication sciences, and 14.3% radiological sciences. 

About 42% of the respondents reported that they were infected with COVID-19, and 6% were hospitalized. Seventy-two percent reported having at least one family member infected. When the students were asked about their perceived CA (PRCA) and self-esteem (RSE), 28.2% (n = 81) reported a high level of communication apprehension, whereas 73.9% (n = 212) reported a high level of self-esteem.

**Figure 1:**
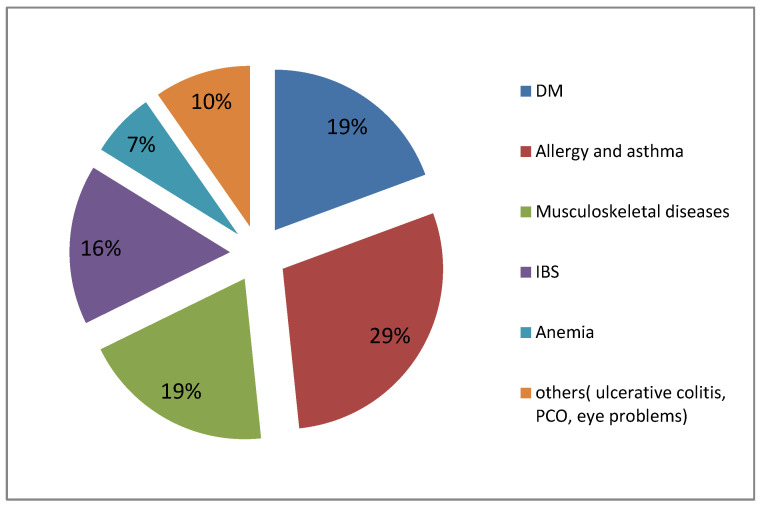
Types of physical issues reported by the sample students (n = 30).

Among them, only 10.5% (30) reported that they suffer from physical health issues as revealed in Table 1; bronchial asthma and allergy were the most reported health problem was 29.0%, as illustrated in Figure 1. 

**Figure 2:**
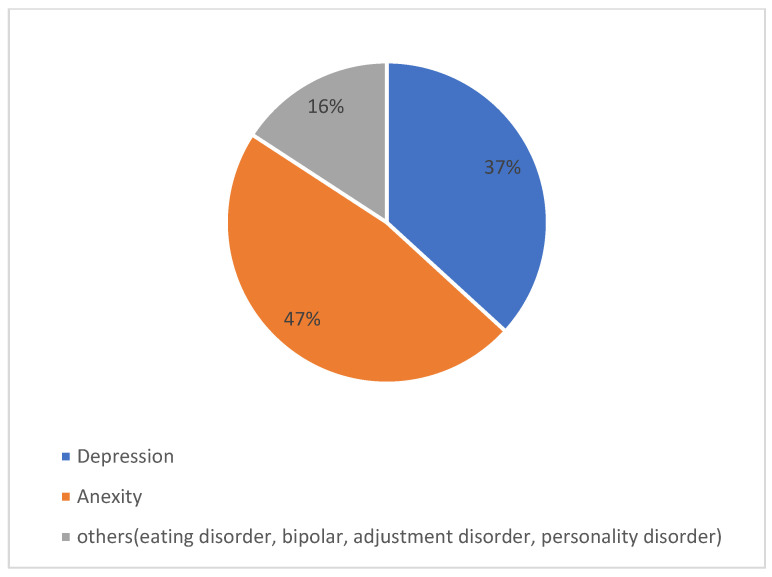
Types of psychological issues reported by the sample students (n = 57).

On the other hand, around 20% had psychological issues. As displayed in Figure 2, the most frequent psychological problem was anxiety, which was reported by 47.4% of the students, followed by depression, which was reported by 37% of them.

Table 2 shows descriptive information about SE, CA and its subscales and internal consistency. The overall PRCA-24 scores for the students from the CHRS ranged from 24 to 119, with the overall mean score being 72.8 ± 16.69, indicating a moderate level of communication apprehension. Scores for each of the four communication apprehension subscales ranged from 6 to 30. Considering the four areas of PRCA24, the mean and standard deviation of the students in the dimensions of group discussion was 18.6 ± 5.04, meetings 18.3 ± 4.94, interpersonal speaking 19.3 ± 4.60, and public speaking 16.7 ± 4.59, respectively. Since a score exceeding 18 is an indication of some degree of communication apprehension (CA), the results of the two dimensions, including group discussions and meetings, showed some degree of apprehension. However, the score for interpersonal speaking indicates a high level of apprehension, whereas public speaking shows a low level of apprehension [34]. On the other hand, RSE ranged from 17 to 32, with overall scores of 24.3 ± 2.14, which indicates a high level of self-esteem. After testing the reliability of the overall PRCA-24, group discussion, meetings, interpersonal, and public speaking using Cronbach’s α coefficient were 0.82, 0.74, 0.84, 0.85, and 0.78, respectively, and Cronbach’s α coefficient for RSE was 0.80, meaning that the tool used was reliable. 

The Mann–Whitney U test was used to determine whether there were differences in PRCA scores between participants according to their personal characteristics (i.e., age groups, marital status, perceived socioeconomic status, GPA, health issues, infection with COVID-19, psychological issues, hospitalization, and infected family members) (Table 3). Only marital status was statistically significantly different between students (U = 470.500, z = −1.85, *p* = 0.05). Thus, the median PRCA was significantly higher in married female students (90; IQR: 36.4) than in single students (73; IQR: 20). Similarly, marital status was statistically significantly different among the students regarding their interpersonal communication (U = 358.000, z = −2.42, *p* = 0.01). The median interpersonal speaking was significantly higher in married female students 22 (IQR: 5.25) than in single students 19 (IQR: 5).

A Kruskal–Wallis H test was conducted to determine if there were differences in overall PRCA, its four subscales, and overall self-esteem regarding their perceived socioeconomic status. Only self-esteem showed significant differences in the median self-esteem between groups, χ^2^ (2) = 6.330, *p* = 0.04. A post hoc analysis was conducted to determine the pairwise comparison between the groups and revealed statistically significant differences, with the mean rank of SE being higher in students from a high social class compared to students from a low social class (168.80 vs. 104.41) with the post hoc Dunn test (Bonferroni *p*-value adjusted = 0.04). Being hospitalized after COVID-19 infection was another factor that created significant differences in their self-esteem, with the median RSE being significantly lower in hospitalized students (23, IQR: 2) (U = 212.50, z = −2.06, *p* = 0.03).

A comparative analysis using Kruskal–Wallis was conducted to determine if there were differences in the overall RSE, PRCA, and its four subscales between sampled participants according to their academic departments. Despite there being no significant differences in the RSE across departments, a statistically significant difference was revealed in the overall PRCA across departments (X^2^ [3] = 8.18, *p* = 0.04). A post hoc Dunn test (Bonferroni *p*-value adjusted = 0.05) confirmed that significant differences exist between health communication sciences and radiological science departments; the mean ranks of PRCA scores were statistically significantly higher in the students in the health communication sciences department than the mean ranks of students in the radiological sciences department (167.8 vs. 121.6).

Similarly, there was a significant difference in public speaking across departments (X^2^ [3] = 14.61, *p* = 0.002). Bonferroni’s post hoc test revealed that the students in the health communication sciences department scored higher (*p* = 0.0003) in public speaking than the students in the rehabilitation sciences department (175.6 vs. 123.2) (Table 4), indicating a lower level of apprehension in public speaking for the former.

In assessing the overall PRCA in the sampled students, 28.2% were categorized as having a high level of communication apprehension, and only 9.8% had a low level of communication apprehension (Table 5). 

**Figure 3:**
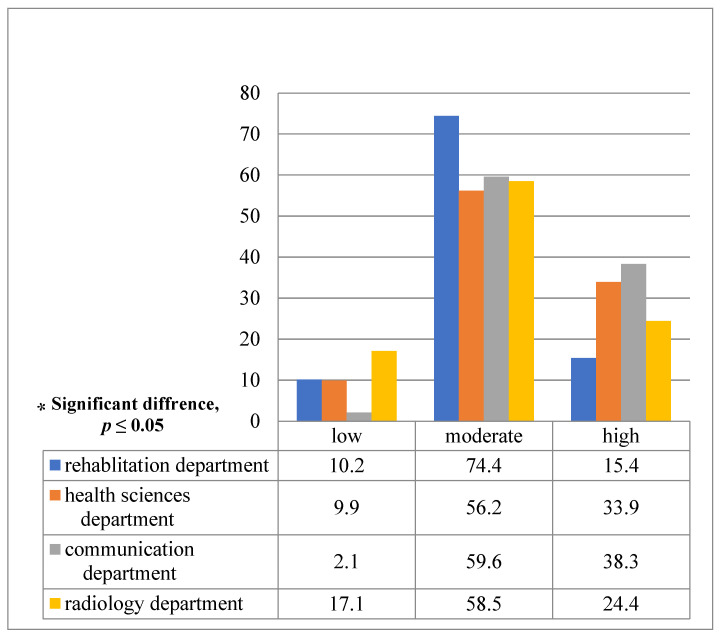
Comparison of the percentage of PRCA categories between students from 4 different academic departments (*p* value = 0.02 *).

After comparing the overall PRCA categories, a statistically significant difference was observed between students from the four different academic departments (χ^2^ [3] = 15.61, *p* = 0.02), with students from the health communication sciences department having the highest communication apprehension (38.3%), while students from the radiological sciences department (17.1%) had the lowest communication apprehension (Figure 3).

The correlation analysis using Spearman rank test showed that there was a negative correlation between GPA and students’ age (r = −0.042, *p* = 0.45): the older the students, the lower their GPA is. Si. Similarly, GPA was negatively correlated with PCRA (r = −0.089, *p* = 0.68) and two of its subscales: Group discussion and Meetings (r = −0.021, *p* = 0.72) (r = −0.022, *p* = 0.70), respectively, meaning that the higher the communication apprehension, the lower was the students GPA score. On the other hand, GPA was slightly positively correlated with self-esteem (r = 0.065, *p* = 0.02), the higher the self-esteem the more the GPA. A significant negative correlation was identified between self-esteem and PCRA (r = −0.195, *p* = 0.001) and two of its subscales: Meetings and public speaking (r = −0.179, *p* = 0.002) (r = −0.089, *p* = 0.002), respectively. Moreover, age was found to be negatively correlated with self-esteem (r = −0.65, *p* = 0.27), and the overall PRCA (r = −0.028, *p* = 0.88). That is, the older the students, the lower their self-esteem and communication apprehension as revealed in Table 6.

To identify factors potentially moderating the effects of SE on communication apprehension and academic achievements, which are measured by GPA, hierarchical moderated regression analysis is used with GPA as the criterion variable, with the independent variables added to the model to determine the moderating effects of such independent variables by the change in the strength of the model. In the first model, all the independent variables were added, including age, social class, marital status, educational level, academic department, physical/health issues, psychological issues, being infected with COVID-19 or having an infected family member, and overall PRCA. The first regression model accounted significantly for about 6% of the variance in students’ GPA (R2 = 0.063, F [2, 287] = 1.02, *p* < 0.05), with only academic departments showing a statistically significant effect (B = 0.14, *p* < 0.05) on GPA. 

Next, the moderator variable was added to the second model while holding other variables constant and showed that self-esteem had no significant effect as a moderating variable for GPA, but it slightly increased the explanatory power of the second regression model by about 2% from the first model (∆R2 = 0.023); consequently, the second model explained only 9% of the variance in GPA (R2 = 0.096, F [2, 287] = 1.03, *p* < 0.05). Finally, the interaction between self-esteem and PRCA was added to the third model and showed that self-esteem, the hypothesized moderating variable, had no significant effect on the relationships between all predictors and GPA. Consequently, they did not have any predictive capacity on GPA (∆R2 = 0.000, R2 = 0.096, F [2, 287] = 0.94, *p* > 0.05) (Table 7).

## 4. Qualitative Analysis

The data was analyzed into two major themes: communication patterns and psychological impact. Furthermore, these two themes were divided into subthemes. The following are the details presented in Table 8.

### 4.1. Theme 1. Communication Patterns

#### 4.1.1. Before Pandemic

*“Yes, actually, I have been a talkative person since elementary school. I took around 100% in competition at King Saud.”*

(R1)

*“Before COVID, I was so confident. So, I used to depend upon my mental skills before. I got many awards by using my mental skills and ability.”*

(R4)

*“For presentations and things that are the group work, I used to be only for leading everything. I used to be so confident. It’s fine with me, you can count on me, it’s OK. I never prepare it early, as long as I know for the scientific parts—I am pretty good.”*

(R2)

*“I was socially active and was not staying at the house with my family.”*

(R6)

*On the other hand, one participant shared, “I’m not a very communicative person”* (R5)*, and one did not report any difference in communication patterns.*

#### 4.1.2. After Pandemic

Communication problems after the pandemic were prominent in their conversation, as follows:

*“It took too long to take part in speech with a lot of people. It was affected at many levels. I’m sorry to speak in Arabic, but it gives me more comfort to use it.”*

(R1)

*“I lose my confidence, so I don’t know how to start [a] new conversation. I feel like, oh, I do this far, I do bad. I lost the basic skills of conversation, maybe.”*

(R4)

*“Now I have to discuss with the leader what I [am] supposed to do for presentations? How long? I never used to do these things before.”*

(R2)

*“I lost myself, but I’m trying to come back to my old habit, so it’s affected me a lot.”*

(R6)

*According to Respondent 5, “After the pandemic, it has helped me to be more aware that we are all in one small world. We should get to know each other better because we are all humans, we like to be in one group, and we like to feel to belong. So now, right now, after the pandemic, I have so many friendships. I even got to communicate with my doctors (teachers).”*

*Respondent 3 added, “Communication [has] become better.”*

### 4.2. Theme 2. Psychological Impact

#### 4.2.1. Psychological Issues

*“Previously, I was stable, [did] not have stress, [did] not have disorganizing thoughts. Right now, everything is changed. I had [a] presentation in the last week that [made] me [so] angry that I was shaking. It’s the first time actually—this is what makes me cry after the presentation. I don’t want to cry, yeah, because it’s difficult for me, the person that [has] a lot of conversations, talks with a lot of people—shaking is not normal for me. This is the first-time presentation after the pandemic.”*

(R1)

*“So, the anxiety level around people, I mean, like, 18 persons and above, I got anxiety.”*

(R5)

*“I’m sensitive now. I face [a stressful] situation; I cry. My teacher suddenly asked me a question—I [was] stuck on one word.”*

(R4)

*“After the pandemic, I used to write [notes] for each thing I have. I’m supposed to say even my comment, even my side thing, or even a good morning doctor. I would like to write down to make sure that I don’t forget anything.”*

(R2)

*“I was crying all the time. I lost a lot of aspects of my personality in this. And this year, I think I lost myself. I didn’t know if I even have doubt with my specialty. Do I* *want to continue to be (…) Is this worth it to continue? This adds depression.”*

(R6)

#### 4.2.2. Anxiety Triggering Event

The respondents replied differently on the subtheme of the most eliciting CA. One participant reported a high anxiety level during the presentation (R1), and another during the class discussion (R4). One respondent replied while conversing with a new acquaintance (R5). In contrast, two respondents reported anxiety during a general conversation (R6 and R2).

#### 4.2.3. Problem Solving

*“So, I prefer to just close [my] mouth and just listen.”*

(R4)

*“I wish I can find the solution for my anxiety.”*

(R5)

*“So, it’s like you know this is the issue I have to find a solution for.”*

(R1)

*At the end of the focus group, they were asked if they wanted to add anything. One of the participants added the following:*

*“I want to mention something about that. I was having a conversation with my colleagues about how they change, and their communications like with the families, with a friend. Most of them, around 10 to 11 (people) in the communication circle—they [are] suffering, [and] they show a significant impact in their patterns of communication, with their family, friends in the university with the doctors (teachers), so perhaps you (professionals) could do something for them. This information could help you in the future, also this seems to spread in all the other students, not only [depending] on us here.”*

(R1)

## 5. Discussion

The study measured the level of self-esteem, CA, and its subscales, and the relationship between them among students at the CHRS during the COVID-19 era. It also investigated the moderating role of self-esteem in the relationship between CA and academic achievement. The results from the study show that almost one in three (28.2%) students who participated in this study reported a high level of communication apprehension. According to the PRCA-24 scores, students had the greatest apprehension in interpersonal speaking (mean = 19.3), which indicates a high level of apprehension. However, the mean scores in group discussions, meetings, and public speaking were found to be 18.6, 18.3, and 16.7, respectively. Based on the PRCA-24 scoring guidelines, the results of the latter subscales indicate some degree of apprehension. This means that students in this study were more apprehensive about communicating with interpersonal speaking than in the other subscales, which might give insight into how interpersonal conversation was affected after the COVID-19 lockdown [37]. During the COVID-19 pandemic, individuals relied more on social media interactions rather than interpersonal communication, and this could be the reason behind the decreased interpersonal speaking [38,39]. 

By comparing departments, significant differences were observed between the apprehension scores of the health communication sciences and radiological sciences departments, where the mean PRCA scores were significantly higher in the former than in the latter. Based on the literature, people with high communication apprehension prefer occupations that require little communication. However, this finding was unexpected since studies in the field of health communication sciences require effective communication skills, which are essential when interacting and communicating with patients with communication disorders [40,41]. Despite a low level of apprehension in public speaking across departments, a significant difference was observed, where the students in the health communication sciences department scored higher in public speaking than the students in the rehabilitation sciences department. The reason for this could possibly be the respondents’ ability to prepare their speech ahead of time. 

Regarding the relationship between CA and age, studies across the literature have shown contradictory findings. Some studies have reported a positive relationship between CA and age [42]; that is, as individuals become older, they tend to have better CA. Whereas other studies have revealed a significant negative relationship between them [43]. However, further studies have shown no relationship between them [44,45].The results of the current study showed a negative correlation between CA and age. That is, the older the students, the lower their CA. The current study also showed that married students had a significantly higher level of CA than single students. A possible explanation could be the burden of household tasks and family responsibilities along with university obligations leading to limited interaction with other students [46,47]. 

Interestingly, the current study found a high level of self-esteem among most of the respondents (73.9%). However, the mean SE score was higher in students from a high social class compared to students from a low social class. Such a finding seems to be consistent with other research, which found that high income is positively related to self-esteem [48]. Further, the finding that the older the students, the higher their self-esteem is consistent with the findings of other studies, including [49] where students tend to have a higher level of self-esteem and stability across their life span. In addition, the median RSE was significantly lower in hospitalized students after a COVID-19 infection. As self-esteem is a strong predictor of depression [50], it might be hypothesized that these students could have developed depression symptoms related to isolation during hospitalization, therefore affecting their self-esteem. Previous studies have also reported several negative feelings that developed because of hospitalization, including depression and low self-esteem [51,52]. This could be another explanation for the findings of the current study. 

The inverse relationship between CA and self-esteem has been well documented in a review conducted by [26]. Thus, as the level of CA decreased, self-esteem increased. The current research noted a strong and negative relationship between overall CA and self-esteem. A comparison of correlation coefficients between subscales of communication apprehension and general self-esteem revealed that meetings and public speaking were strongly and negatively associated with self-esteem. 

On the other hand, group discussion and interpersonal speaking were strongly and positively associated with self-esteem. Thus, students with high self-esteem tend to have anxiety communicating in small groups and interpersonal communication. This interesting finding could be explained by the precautions forced by the COVID-19 pandemic. Since students were instructed to remain isolated or socially distanced during the pandemic, apprehension in interpersonal communication and small-group discussions could be brought on by the pandemic and thus affect the students’ communication patterns and habits. 

Regarding academic achievement, as assessed by GPA, the findings revealed a nonsignificant negative correlation between academic achievement and age; thus, older students have weaker academic achievement throughout the academic levels. This is consistent with other studies [53,54]. Regarding the relationship between CA and academic achievement, a meta-analysis study found that CA has a small negative correlation with academic achievement [30]. Hence, the academic achievement of students with higher CA can only be affected if their CA prevents them from asking questions and participating in class exercises. The current study found a non-significant negative correlation between CA and overall GPA. This suggests that CA may not have a negative effect on academic performance, at least for the present sample, and a similar finding was reported by Blume et al. [55]. 

On the other hand, high self-esteem could play an important role in and strengthen the prediction of academic achievement. This conclusion was drawn based on the assumption that low self-esteem causes a lack of interest in achieving high academic achievements [31]. In the current study, GPA was significantly positively correlated with self-esteem. However, despite such a strong correlation and the high level of self-esteem among the students, we found no moderating effect of self-esteem on the association of communication apprehension with academic achievement, as assessed by GPA. Based on this result, it can be suggested that having a high value (self-esteem) does not have a preventive function over the negative effect of CA on GPA. However, based on other studies, other traits could serve as predictors of GPA, including intelligence, personality, motivational traits, self-efficacy, stress, or first-year GPA [56,57,58,59]. CA was found to have a high score variable among students; therefore, a focus group was conducted for a more detailed investigation.

The focus group revealed firsthand information to comprehend issues related to CA. COVID-19 has had a massive impact on all dimensions of life, including communication, which is crucial for the expression of self, feelings, ideas, knowledge sharing, and the learning process [60]. Among the focus group participants, four respondents expressed that they were good at communication, socially active, and confident before the pandemic. 

On the second sub-theme of communication patterns after the pandemic, four participants shared that they had been experiencing various communication problems, including initiating speech, nervousness, and lack of confidence in resuming back to regular classes. Previous studies have also confirmed the adverse impact of COVID-19 pandemic on communication skills [5]. Only two participants reported improved communication and social skills. These respondents were either not very communicative or did not experience any differences in communication. These responses might be justified by having high resilience or compensating for a prolonged period of isolation due to a pandemic [61,62].

The negative impact of a pandemic is devastating. COVID-19 not only affected communication, but also left a scar on the mental health and well-being of students. Some of these post-pandemic effects have been revealed in previous studies [63]. However, some long-lasting impacts will take time to unveil. This study asked what difficulties students were facing in communication after the COVID-19 pandemic when participating in class discussions, conversing with a new acquaintance, or giving a speech/presentation. The CHRS female students expressed the psychological issues, including stress, anxiety, stuttering, apprehensions, depression, and insomnia, that they were experiencing after the pandemic. Former studies have confirmed nervousness, uneasiness, and vulnerability too among female students while communicating in front of the class [3]. Which will decrease their self-confidence and undermine their feeling of security. Most of the issues discussed were self-identified by the students. However, they show a need for support, empathetic listening, and psychological help [62].

Knowing what kinds of efforts people make to overcome the psychological fear cultivated by the pandemic is essential. Any stressful situation prepares people for various reactions [17]. Therefore, among the six focus group members, one came up with a flight response and two with a fight response. 

It can be concluded that COVID-19 has caused many psychological issues and has had a negative impact upon communication. However, progressively, the young female students of CHRS are trying to uplift their morale with courage and by sharing their worries.

## 6. Study Limitations and Future Implications

The study provides insights into the levels of self-esteem, CA, and academic performance among Saudi female students during the COVID-19 era. One of the practical implications that was supported by the results is related to CA. There is a need to conduct a series of workshops, training sessions or counseling sessions for the students to overcome CA. Moreover, instructors can also be trained to handle stress and CA during various educational activities including class presentations and discussions. Furthermore, during an outbreak of emergency situations, such as the COVID-19 lockdown, educational interventions are recommended to focus on training students with low self-esteem and those with poor communication to enhance their communication skills to avoid any suffering that negatively affects their psychological wellbeing. However, some limitations should be mentioned. Given that the university admits only female students, the study included only female students, which affected the study’s generalizability. Future research might examine the relationship between self-esteem and CA in both genders. Furthermore, the cross-sectional design does not support the causal relationship between exposure and outcome; thus, a longitudinal study design is required to determine the cause-and-effect relationship. 

Moreover, due to the sudden nature of the COVID-19 pandemic, it was not possible to conduct a longitudinal study to investigate the impact of COVID-19 on students’ self-esteem and CA, and to investigate the change in them that could be caused by COVID-19. A further limitation is using a self-reporting questionnaire, which might come with its limitation in terms of reporting bias; however, we used a mixed method to overcome that limitation in self-reporting by implementing a focus group to interview the students and understand these factors. 

## 7. Conclusions

The study examined the level of self-esteem, CA, and academic performance among Saudi female students that; students had the greatest apprehension in Interpersonal Speaking, which indicates a high level of concerns. Students relied more on social interactions rather than interpersonal communication, which can be a reason behind the decreased interpersonal speaking. The students’ interpersonal speaking showed a high level of apprehension, whereas public speaking shows a low level of apprehension. The findings proved the higher the communication apprehension, the lower was the students GPA score. On the other hand, GPA was slightly positively correlated with self-esteem. Self-esteem proved no effect of predictors and the students’ academic performance. 

To conclude, endeavors need to be made to enhance students’ communication and self-esteem through educational interventions to leave a powerful effect on students’ psychological well-being and to improve their resilience to face difficulties they may encounter. 

## Figures and Tables

**Table 1:** Descriptive stats for Personal characteristics of the respondent students.

Characteristics	Responses	No (%)
Age group	<21	118 (41.1)
	≥21	169 (58.9)
	20.81 ± 1.4	
Marital status	Single	281 (97.9)
	Married	6 (2.1)
Perceived socioeconomic status	Low	16 (5.6)
	Middle	243 (84.7)
	High	28 (9.8)
Level	4	83 (28.9)
	6	95 (33.1)
	8	100 (34.8)
	Higher	9 (3.1)
Department	Health sciences	78 (27.2)
	Rehabilitation	121 (42.2)
	Communication	47 (16.4)
	Radiology	41 (14.3)
GPA	<4.5	125 (43.6)
	≥4.5	162 (56.4)
	4.48 ± 0.32	
Physical health issues	Absent	257 (89.5)
	Present	30 (10.5)
Psychological issues	Absent	230 (80.1)
	Present	57 (19.9)
Infected with COVID-19	No	168 (58.5)
	Yes	119 (41.5)
Hospitalized	No	112 (94.1)
	Yes	7 (5.9)
Family members infected	No	80 (27.9)
	Yes	207 (72.1)
Total		287 (100.0)

GPA: Students’ Grade Point Average; PRCA: Personal Report Communication Anxiety; RSE: Rosenberg Self-Esteem.

**Table 2:** Descriptive and Internal consistency of the Self-esteem and Communication apprehension and its subscales.

Scales/Subscales	Items	M	SD	Mdn	IQR	MIN–MAX	Cronbach’s α Coefficient
GD	6	18.6	5.04	18.6	6	6–30	0.74
Ms	6	18.3	4.94	18	7	6–30	0.84
IP	6	19.3	4.60	19	5	6–30	0.85
PS	6	16.7	4.59	17	5	6–30	0.78
PRCA	24	72.8	16.69	73	21	24–119	0.82
RSE	10	24.3	2.14	25	3	17–32	0.80

M: mean; SD: standard deviation; Mdn = Median; IQR = Interquartile Range; GD: Group Discussion; Ms: Meetings; IP: Interpersonal; PS: Public Speaking; PRCA: Personal Report Communication Anxiety; RSE: Rosenberg Self-esteem.

**Table 3:** Comparing personal characteristics of respondents with PRCA and RSE (N = 287).

Characteristics	Responses	PRCA *Mdn (IQR)*	SUBSCALES				
			GD *Mdn (IQR)*	Ms *Mdn (IQR)*	PS *Mdn (IQR)*	PI *Mdn (IQR)*	RSE
Age groups (y)	<21	73 (17.3)	19 (5.5)	18 (6)	17 (5)	19 (5)	25 (3)
	≥21	73 (22)	19 (7)	18 (7.5)	17 (6)	19 (5)	24 (3)
Test		9722.000 (−0.36)	9766.000 (−0.297)	9480.500 (−0.712)	9017.000 (−1.385)	9916.500 (−0.079)	9292.000 (−0.996)
* p * Value		0.719	0.766	0.477	0.166	0.937	0.319
Marital status	Single	73 (20)	19 (6)	18 (6.5)	17 (5.5)	19 (5)	25 (3)
	Married	90 (36.4)	25 (12.3)	24(12.25)	18.5 (8)	22 (5.25)	26 (3)
Test		470.500 (−1.85) *^U^*	484.0000 (−1.8)	524.500 (−1.59)	573.500 (−1.35)	358.000 (−2.42)	525.500 (−1.6)
* p * Value		0.05 *	0.073	0.112	0.179	0.016 *	0.11
Perceived socioeconomic status	Low	68 (31)	16 (3.75)	16.5 (10)	17 (10.75)	17 (4.75)	24 (3)
	Intermediate	73 (19)	19 (6)	18 (7)	17 (5)	19 (4)	25 (3)
	High	72 (21.5)	18 (8.5)	18 (9.25)	16.5 (7.25)	19 (8.5)	25 (2)
Test		2.612 (2) *^H^*	2.860 (2) *^H^*	0.976 (2) *^H^*	1.507 (2) *^H^*	3.098 (2) *^H^*	6.330 (2) *^H^*
* p * Value		0.271	0.239	0.614	0.471	0.213	0.042 *
GPA		72 (20.25)	19 (7)	18 (8)	17 (6)	19 (5)	25 (2.25)
		73 (19)	19 (6.5)	18 (6)	17 (5)	19 (5.5)	24 (3)
Test		9461.500 (−0.95) *^U^*	9746.500 (−0.545)	9435.500 (−0.993)	9680.000 (−0.641)	9859.000 (−0.383)	9882.000 (−0.354)
* p * Value		0.341	0.586	0.321	0.522	0.702	0.723
Physical/health issues	Absent	73 (19)	19 (6)	18 (7)	17 (5.5)	19 (5)	25 (3)
	Present	73 (19.75)	18.5 (5.7)	18 (8.25)	16.5 (4.5)	19.5 (6.25)	24 (3)
Test		3733.000 (−0.28)	3747.500 (−0.25)	3696.500 (−0.37)	3452.500 (−0.94)	3753.000 (−0.24)	3331.500 (−1.24)
* p * Value		0.777	0.802	0.711	0.347	0.812	0.217
Psychological issues	Absent	73 (19)	19 (6.25)	18 (7)	17 (5.5)	19 (5)	25 (3)
	Present	73 (23)	18 (7.5)	18 (7.5)	17 (4.5)	19 (5)	24 (3.5)
Test		5816.500 (−1.32)	5614.500 (−1.682)	5857.000 (−1.249)	6073.500 (−0.862)	5654.500 (−1.611)	6068.500 (−0.880)
* p * Value		0.188	0.093	0.211	0.389	0.107	0.379
Infected with COVID-19	No	73 (19.5)	19 (6.75)	18 (6)	17 (5)	19 (5)	25 (2)
	Yes	73 (23)	18 (7)	18 (7)	17 (7)	19 (5)	25 (2)
Test		9970.500 (−0.04)	9593.500 (−0.583)	9501.000 (−0.718)	9191.000 (−1.167)	9805.500 (−0.276)	9384.000 (−0.897)
* p * Value		0.971	0.560	0.473	0.243	0.783	0.370
Hospitalized	No	73 (23)	18 (7)	18 (8)	17.5 (7)	20 (5.75)	25 (2)
	Yes	73 (7)	19 (2)	18 (5)	17 (5)	19 (1)	23 (2)
Test		366.500 (−0.29)	371.500 (−0.232)	333.000 (−0.669)	346.000 (−0.522)	334.500 (−0.651)	212.500 (−2.060)
* p * Value		0.773	0.816	0.504	0.602	0.515	0.039 *
Infected Family members:	No	73 (15.5)	18 (5.75)	18 (6.5)	17 (4)	19 (3)	25 (3)
	Yes	73 (22)	19 (7)	18 (7)	17 (6)	19 (5)	25 (3)
Test		8123.500 (−0.25)	8188.500 (−0.15)	7921.500 (−0.57)	7893.000 (−0.62)	8141.000 (−0.22)	8001.000 (−0.45)
* p * Value		0.804	0.884	0.568	0.538	0.825	0.653

* Significance difference (*p* ≤ 0.05); *^U^* = Mann–Whitney test; *^H^* = Kruskal–Wallis test; *Mdn* = Median; IQR = interquartile range; B: Post hoc comparisons were conducted using the Bonferroni-corrected post hoc *t* tests; GPA: Students’ Grade Point Average; GD: Group discussion; Ms: Meetings; IP: Interpersonal; PS: Public Speaking; PRCA: Personal Report Communication Apprehension; RSE: Rosenberg Self-Eesteem.

**Table 4:** Comparing PRCA and RSE in students from different departments (N = 287).

Characteristics	Responses	PRCA *Mdn (IQR)*	Subscales				RSE *Mdn (IQR)*
			GD *Mdn (IQR)*	Ms *Mdn (IQR)*	PS *Mdn (IQR)*	PI *Mdn (IQR)*	
Department	RehabS (n = 78)	73 (13.3)	19 (5)	18 (5)	17 (5)	19 (4)	24 (2)
	HS (n = 121)	74 (24.5)	19 (8.5)	18 (7.5)	17 (6)	19 (6)	25 (3)
	RS (n = 41)	69 (24.5)	17 (6)	18 (9)	16 (6.5)	18 (7)	24 (3)
	HCS (n = 47)	75 (12)	19 (7)	18 (7)	18 (4)	20 (5)	25 (2)
Test		8.18 *^H&B^*	5.165 *^H^*	4.35^*H*^	14.61^*H&B*^	4.58 *^H^*	6.51 *^H^*
* p * Value		0.04 *	0.16	0.24	0.002 *	0.21	0.09
Group differences		HCS > RS 0.05 *			HCS > RS 0.003 *		

* Significance difference (*p* ≤ 0.05); *^H^* = Kruskal–Wallis test; *Mdn* = Median; IQR = interquartile range; *^B^*: Post hoc comparisons were conducted using the Bonferroni-corrected post hoc *t* tests; GD: Group discussion; Ms: Meetings; IP: Interpersonal; PS: Public Speaking; PRCA: Personal Report Communication Apprehension; RSE: Rosenberg Self-esteem; RehabS: Rehabilitation Sciences; HS: Health Sciences; HCS: Health Communication Sciences; RS: Radiological Sciences.

**Table 5:** The overall PRCA in the sampled students.

Characteristics	Responses	No (%)
PRCA	Low	29 (9.8)
	Average	178 (62.0)
	High	81 (28.2)
RSE	Low	75 (26.1)
	High	212 (73.9)

PRCA: Personal Report Communication Anxiety; RSE: Rosenberg Self-esteem.

**Table 6:** Spearman Correlation between GPA with Rosenberg SES, Communication anxiety, GPA among study respondents.

	Correlation Matrix for the Study Variables							
Variables	GPA	Age	RSE	PRCA	GD	MS	IP	PS
GPA	1							
Age	−0.042	1						
RSE	0.065	−0.65	1					
PRCA	−0.089	−0.028	−0.195 **	1				
GD	−0.021	0.011	0.178 **	0.859 **	1			
MS	−0.022	−0.025	−0.179 **	0.910 **	0.738 **	1		
IP	0.039	0.036	0.144 *	0.841 **	0.648 **	0.707 **	1	
PS	0.067	−0.064	−0.165 **	0.752 **	0.523 **	0.643 **	0.531 **	1

* Correlation is significant at 0.05 level; ** Correlation is significant at 0.01 level GPA: Students’ Grade Point Average; GD: Group discussion; Ms: Meetings; IP: Interpersonal; PS: Public Speaking; PRCA: Personal Report Communication Anxiety; RSE: Rosenberg Self-esteem.

**Table 7:** Hierarchical moderating regression analysis for the predictors of GPA among study respondents.

	Variables	Model 1				Model 2				Model 3			
		B	Β	T	Sig.	B	Β	T	Sig.	B	β	T	Sig.
Constant		4.270		11.986	0.000	4.012		9.516	0.000	4.010		9.490	0.000
Predictors	Age	−0.005	−0.022	−0.379	0.705	−0.004	−0.018	−0.307	0.759	−0.004	−0.019	−0.312	0.756
	Marital status	0.122	0.055	0.905	0.366	0.115	0.052	0.857	0.392	0.116	0.052	0.858	0.392
	Social class	0.034	0.042	0.707	0.480	0.026	0.032	0.532	0.595	0.026	0.032	0.533	0.595
	Department	0.047	0.144	2.379	0.018	0.046 *	0.143	2.358	0.019 *	0.046	0.143	2.354	0.019 *
	Physical/health issues	0.036	0.035	0.586	0.558	0.042	0.040	0.676	0.500	0.041	0.040	0.662	0.509
	Psychological issues	0.039	0.048	0.809	0.419	0.042	0.053	0.878	0.381	0.042	0.052	0.871	0.385
	Infected with COVID-19	−0.069	−0.106	−1.669	0.096	−0.070	−0.108	−1.705	0.089	−0.070	−0.107	−1.683	0.094
	Relative infected with COVID-19	0.017	0.024	0.387	0.699	0.018	0.026	0.412	0.681	0.018	0.025	0.403	0.688
	PRCA	0.004	0.011	0.185	0.854	5.518	0.000	0.003	0.998	0.000	0.000	−0.007	0.995
Moderators	RSE					0.010	0.070	1.144	0.254	0.011	0.071	1.151	0.251
Interaction	PRCA × RSE									−0.003	−0.009	−0.148	0.883
Model Statistics	F	1.020 *				1.034				0.944			
	R^2^	0.063				0.096				0.096			
	∆R^2^	0.063				0.023				0.000			

* *p* ≤ 0.05 is significance; B: unstandardized beta “regression coefficient”; β: standardized beta; F: ANOVA; ∆R2: R2 change; R2: coefficient of determination; PRCA: Personal Report Communication Anxiety; RSE: Rosenberg Self-Esteem; a. Predictors: (Constant), age, SES, Marital status, level, department, physical/health issues, psychological issues, infected with COVID-19, infected family member, PRCA; b. Predictors: (Constant), age, SES, Marital status, level, department, physical/health issues, psychological issues, infected with COVID-19, infected family member, PRCA, RSE; c. Predictors: (Constant), age, SES, Marital status, level, department, physical/health issues, psychological issues, infected with COVID-19, infected family member, PRCA, RSE, moderator; d. Dependent Variable: GPA.

**Table 8:** Summary of themes.

	Themes	Sub-Themes	Description	Example/Main points
1	Communication patterns	Before pandemic	The quality of expressive communication in general before pandemic.	“I had been a talkative person”.
		After pandemic	Difficulties in Communication pattern after pandemic.	“I lost the basic skills of conversation maybe”.
2	Psychological impact	Psychological Issue	Psychological issue in communication after the COVID-19 pandemic.	“I am sensitive now”.
		Anxiety triggering event	Circumstance causing nervousness and stress.	Presentation, class discussion, conversing with a new acquaintance, general conversation.
		Problem-solving	The problem-solving approaches used by the participants.	“I wish I can find the solution for my anxiety”.

## Data Availability

Data will be available on request.
